# Oxidant-Free Thiol–Ene Grafting of Granular Activated Carbon for Mercury(II) Adsorption from Aqueous Solution and Synthetic Gas Condensate

**DOI:** 10.1021/acsomega.6c09402

**Published:** 2026-10-26

**Authors:** Aphinan Saengsrichan, Jakkapop Phanthasri, Supawadee Namuangruk, Poobodin Mano, Suttipong Wannapaiboon, Doan Pham Minh, Sanchai Kuboon, Saran Youngjan, Teera Butburee, Issaraporn Rakngam, Panot Krukkratoke, Gopinathan Sankar, Nurak Grisdanurak, Suthirat Kittpongvises, Rattabal Khunphonoi, Pongtanawat Khemthong, Pummarin Khamdahsag

**Affiliations:** † International Program in Hazardous Substance and Environmental Management, College of Interdisciplinary and Integrative Studies, 26683 Chulalongkorn University, Bangkok 10330, Thailand; ‡ Center of Excellence on Hazardous Substance Management (HSM), Chulalongkorn University, Bangkok 10330, Thailand; § National Nanotechnology Center (NANOTEC), National Science and Technology Development Agency (NSTDA), Pathum Thani 12120, Thailand; ∥ Synchrotron Research and Applications Division, Synchrotron Light Research Institute (Public Organization), Nakhon Ratchasima 30000, Thailand; ⊥ Centre RAPSODEE, IMT Mines Albi, UMR CNRS 5302, Université de Toulouse, 81013 Albi, Cedex 9, France; # Sustainable Environment Research Institute, Chulalongkorn University, Bangkok 10330, Thailand; ∇ Department of Chemistry, 4919 University College London, London WC1H 0AJ, United Kingdom; ○ Center of Excellence in Environmental Catalysis and Adsorption, Faculty of Engineering, Thammasat University, Pathum Thani 12121, Thailand; ◆ Department of Environmental Engineering, Faculty of Engineering, 26684 Khon Kaen University, Khon Kaen, Nai Mueang 40002, Thailand; ¶ Hub of Waste Management for Sustainable Development, Center of Excellence on Hazardous Substance Management, Chulalongkorn University, Bangkok 10330, Thailand

## Abstract

Mercury removal from aqueous and hydrocarbon media requires sorbents that combine strong binding affinity with sufficient pore accessibility and mass transfer. In this study, granular activated carbon (GAC) was treated with 1,2-ethanedithiol through an oxidant-free, thermally initiated thiol–ene-type radical route to produce sulfur-functionalized GAC (S-GAC) with different sulfur loadings. The sulfur content increased from 3.38 to 15.07 wt %, while the BET specific surface area decreased from 859 to 25 m^2^ g^–1^, indicating progressive pore occupation and blockage. At low initial Hg­(II) concentration, sulfur functionalization enhanced Hg­(II) removal in both aqueous solution and synthetic gas condensate, with 10–30%S-GAC achieving very low residual Hg­(II) concentrations. At elevated aqueous Hg­(II) concentrations, pristine GAC exhibited faster initial uptake and approached a plateau earlier, whereas S-GAC showed slower but sustained adsorption. At approximately 40 mg L^–1^ Hg­(II) concentration, 5%S-GAC and 10%S-GAC ultimately achieved slightly higher observed uptakes than pristine GAC, while at approximately 120 mg L^–1^ pristine GAC retained the highest uptake over the monitored period. These results show that the effect of sulfur functionalization depends on Hg­(II) loading, contact time, and the balance between Hg–S affinity and pore accessibility. XPS, Hg L_3_-edge XAS, and DFT calculations were consistent with sulfur-containing Hg coordination environments and more favorable interaction near sulfur-rich edge configurations. Sulfur functionalities remained strongly retained over pH 2–10, although regeneration performance decreased with repeated cycles. Overall, Hg­(II) capture reflects the combined effects of sulfur-site affinity, pore accessibility, mass transfer, Hg­(II) loading, and the treatment matrix.

## Graphical abstract

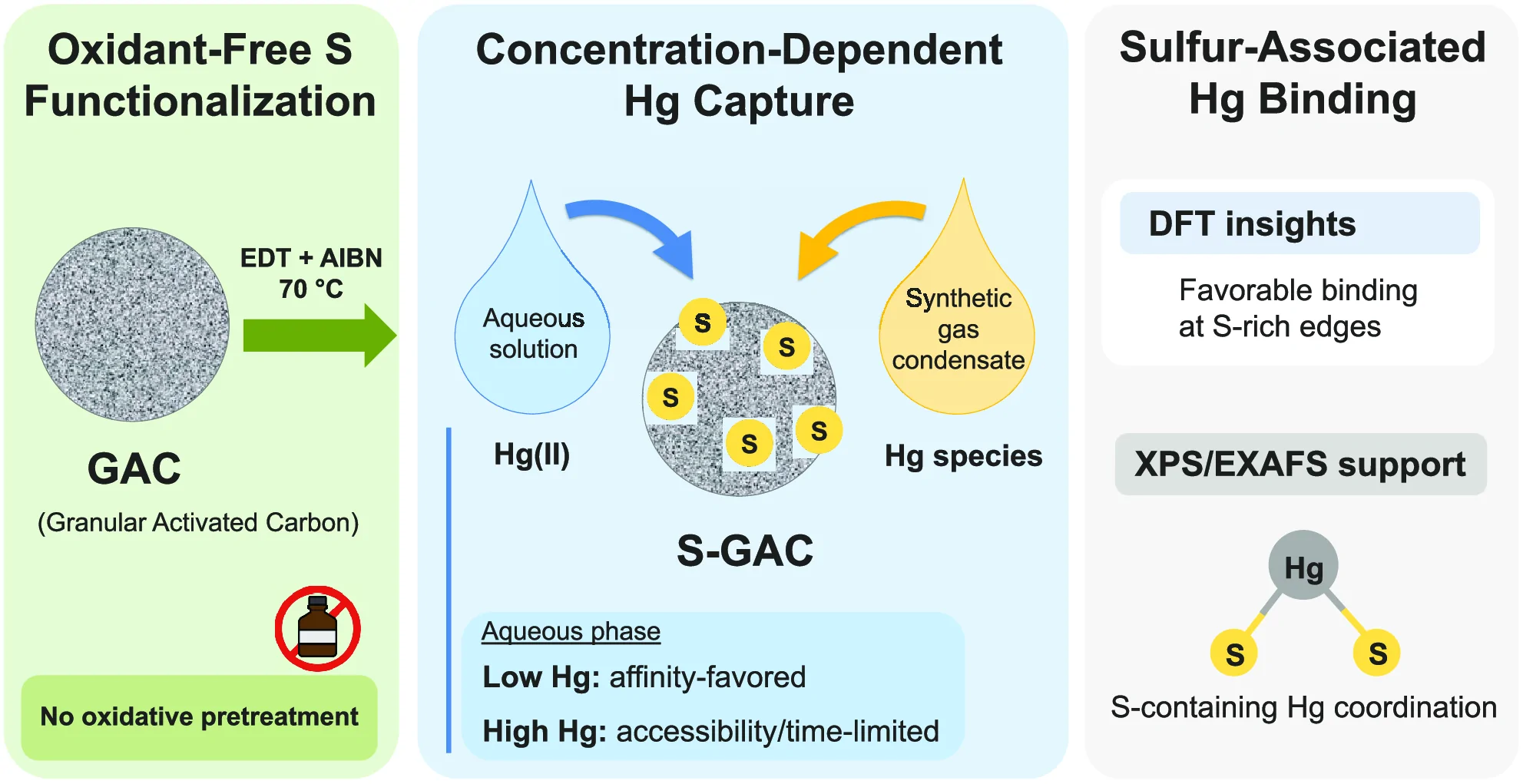

## 1 Introduction

Mercury is a persistent contaminant encountered in natural gas processing, condensate streams, and associated aqueous phases. Depending on the process conditions and phase partitioning, mercury may occur as a reactive ionic species in aqueous fractions or as a solvated species in hydrocarbon media.^1^ Its presence poses operational concerns because mercury can induce liquid-metal embrittlement of aluminum alloys used in cryogenic heat exchangers, potentially leading to equipment damage and process failure.^2–5^ In addition to these operational hazards, mercury is a potent neurotoxin with far-reaching consequences for human health^6^ and ecosystem stability,^7^ motivating the need for stringent control across natural gas processing, petrochemical operations, and associated wastewater streams.^8^ These concerns create a need for sorbent materials capable of retaining mercury under both aqueous and hydrocarbon conditions.

Activated carbon (AC) is widely used for mercury removal because of its high surface area, commercial availability, mechanical stability, and suitability for granular treatment systems.^9,10^ However, pristine-activated carbon generally lacks a sufficient density of soft donor functional groups that selectively coordinate Hg­(II). Surface oxidation is typically used to introduce reactive oxygen-containing groups prior to further functionalization, but such treatments may require concentrated oxidants, generate secondary waste, and alter the porous carbon structure.^11–14^ Direct incorporation of sulfur-containing functionalities therefore represents an alternative approach for increasing mercury affinity while avoiding corrosive oxidative pretreatment.

Sulfur-containing ligands, particularly thiol and sulfide groups, interact strongly with mercury through soft acid–soft base interactions and can form stable Hg–S coordination environments.^15^ A range of sulfur-modified materials, including sulfurized biochars, thiol-functionalized activated carbons, metal–organic frameworks, and carbon-based nanomaterials, have consequently been investigated for Hg­(II) adsorption.^16–21^ However, most previous studies have focused on aqueous systems, whereas fewer have examined mercury capture in hydrocarbon media or compared adsorption behavior across matrices of substantially different polarities. In addition, increasing the total sulfur content does not necessarily increase the number of accessible binding sites because dense functionalization may obstruct pores, restrict mass transfer, or create sterically hindered sulfur environments.^8,22,23^ Understanding the balance between sulfur-site density and accessibility is therefore important for designing sulfur-modified granular adsorbents.

Thiol–ene chemistry provides a potentially useful framework for introducing sulfur-containing functionalities onto unsaturated carbon sites through thermally initiated radical addition under relatively mild conditions.^23–26^ When applied to heterogeneous carbonaceous materials, however, the resulting surface chemistry may include a distribution of 1,2-ethanedithiol (EDT)-derived C–S, S–S, and residual thiol environments rather than a single well-defined molecular structure. Thermally initiated thiol–ene-type treatments may nevertheless enable one-pot sulfur functionalization without prior oxidative activation.^23–26^ Although thiol-based modification has been applied to several carbonaceous materials,^27–34^ the relationships among sulfur loading, pore accessibility, mercury uptake, and local Hg–S binding environments in granular activated carbon remain insufficiently understood, particularly when aqueous and hydrocarbon matrices are compared under similar adsorption conditions.

In this study, 1,2-ethanedithiol was introduced onto granular activated carbon through an oxidant-free, thermally initiated thiol–ene-type radical treatment to prepare sulfur-functionalized granular activated carbon with different sulfur loadings. The materials were evaluated for Hg­(II) adsorption from aqueous solution and synthetic gas condensate to examine how sulfur loading, pore accessibility, Hg­(II) concentration, and the treatment matrix collectively influence the adsorption performance. Particular attention was given to the balance between the strong affinity of sulfur-containing sites for Hg­(II) and the progressive loss of accessible porosity associated with increasing sulfur loading. The relationships among sulfur content, textural properties, and adsorption behavior were examined, and X-ray photoelectron spectroscopy, Hg L_3_-edge X-ray absorption spectroscopy, and density functional theory calculations were used to provide molecular-level insight into the Hg–S coordination environments associated with adsorption.

## 2 Experimental Section

### 2.1 Chemicals

Coconut-shell granular activated carbon (GAC; 2.36–4.75 mm), produced by pyrolysis and steam activation, was obtained from CarboKarn (Thailand). 1,2-Ethanedithiol (EDT, 99%) and azobis­(isobutyronitrile) (AIBN, 98%) were purchased from TCI America. Toluene (99.5%), *n*-hexane (99%), and *n*-heptane (95%) were obtained from RCI Labscan (Thailand). Ethanol (98%) and hydrochloric acid (37%) were purchased from Merck (Thailand), sodium hydroxide (97%) from KemAus (Australia), and mercury­(II) chloride (99.5%) from Loba Chemie (India). Deionized water (18.2 MΩ cm) was used throughout. All of the chemicals were used as received.

### 2.2 Synthesis of S-GAC

Sulfur functionalities were introduced onto GAC through an oxidant-free, thermally initiated radical thiol–ene-type treatment using EDT. For each synthesis batch, the GAC (5.0 g) was immersed in ethanol (20 mL) in a capped Duran bottle. AIBN was added at 1 wt % of the sulfur mass supplied by EDT, and the suspension was stirred at 70 °C for 10 min in a silicone-oil bath. EDT was then added in amounts corresponding to nominal sulfur-to-GAC mass ratios of 5, 10, 15, and 30 wt %. The mixture was stirred at 100 rpm and 70 °C for 8 h. The resulting S-GAC was recovered by vacuum filtration, washed three times by soaking in 30 mL of ethanol at 40 °C for 5 min per cycle, and dried under vacuum (−0.1 bar) at 40 °C for 12 h. The products were denoted as 5%S-GAC, 10%S-GAC, 15%S-GAC, and 30%S-GAC according to their nominal sulfur-to-GAC mass ratios.

### 2.3 Characterization of S-GAC

Raman spectra were recorded using a 532 nm laser over 10–1800 cm^–1^ to identify carbon structural features and sulfur-related vibrations (Horiba Scientific, Japan). The total sulfur contents were determined in triplicate using a CHNS elemental analyzer (LECO 628 Series, USA) after the samples were dried at 80 °C; a sulfur-containing coal reference material (1.16 ± 0.06% S) was used for quality control. Surface morphology and elemental distributions were examined by FE–SEM–EDS (Hitachi SU8230, Japan) at an accelerating voltage of 15 kV after the samples were mounted on carbon tape and sputter-coated with gold.

Thermal behavior was analyzed by thermogravimetric analysis (Mettler Toledo, Switzerland) in air at a heating rate of 10 °C min^–1^. The textural properties were determined from N_2_ adsorption–desorption isotherms at 77 K using a Quantachrome NOVA e-Series analyzer (USA). The samples were degassed under vacuum at 150 °C for 12 h before analysis, and the specific surface area was calculated using the BET method. The point of zero charge, pH_pzc_, was determined over an initial pH range of 2–10, adjusted using 0.1 M HCl or NaOH, following the method of Chikri et al.^35^

Surface elemental compositions and chemical states were analyzed by X-ray photoelectron spectroscopy using a PHI5000 VersaProbe II instrument (ULVAC-PHI, Japan) with monochromatic Al Kα radiation (*h*ν = 1486.6 eV). Survey spectra were collected at a pass energy of 117.4 eV and analyzed using CasaXPS. The local coordination environment of adsorbed Hg­(II) was examined by Hg L_3_-edge X-ray absorption spectroscopy at Beamline 7.2 W, SLRI, Thailand. XANES and EXAFS data were processed using ATHENA and ARTEMIS, respectively, to evaluate the Hg oxidation state, coordination number, and bond distance.

### 2.4 Mercury Adsorption

Hg­(II) adsorption by pristine GAC and S-GAC was evaluated in aqueous solution and synthetic gas condensate using batch experiments. Hg­(II) stock solutions were prepared from HgCl_2_, and working solutions were freshly prepared before each experiment. Adsorption experiments were conducted in 1000 mL beakers at 25 ± 2 °C and 100 rpm using an adsorbent dosage of approximately 1.0 g L^–1^ and a working volume of 800 mL. For aqueous experiments, the initial pH was adjusted to 6.0 ± 0.2 using 0.1 M HCl or NaOH.^36^ A synthetic gas condensate was prepared by mixing *n*-hexane, *n*-heptane, toluene, and *p*-xylene at 35:35:15:15 (v/v/v/v). The resulting mixture contained 70 vol % saturated hydrocarbons and 30 vol % aromatic compounds and was used as a condensate-like hydrocarbon matrix for Hg­(II) adsorption experiments.^36,37^

Time-dependent adsorption at low initial Hg­(II) concentration was evaluated using approximately 1 mg L^–1^ Hg­(II) in both aqueous solution and synthetic gas condensate. Aqueous samples were collected over 12 h, whereas condensate samples were collected over 6 h. To further examine the adsorption behavior under higher Hg­(II) loadings and prolonged contact times, additional aqueous-phase experiments were conducted at initial Hg­(II) concentrations of approximately 40 and 120 mg L^–1^. These experiments were monitored for up to 288 h (12 days) and 576 h (24 days), respectively, using the same adsorbent dosage, solution volume, temperature, agitation rate, and pH conditions described above. At predetermined contact times, aqueous and condensate samples were withdrawn and filtered through 0.22 μm nylon syringe filters to remove suspended adsorbent particles. Residual Hg­(II) concentrations in aqueous samples were quantified by inductively coupled plasma-optical emission spectroscopy (ICP-OES; PlasmaQuant 9100 Series, Analytik Jena, Germany). Mercury concentrations in synthetic gas condensate were determined using a DMA-80 evo direct mercury analyzer (Milestone Srl, Italy) following US EPA Method 7473. Time-dependent adsorption experiments were performed in duplicate, whereas stability and reusability tests were performed in triplicate.

### 2.5 Density Functional Theory Calculations

DFT calculations were performed to elucidate the Hg­(II) binding configurations of S-GAC. Cluster models representing sulfur-grafted GAC with isolated (S-GAC1) and high-density (S-GAC2) sulfur motifs were constructed (Figure 1). Adsorption of HgCl_2_ and HgCl^+^ species was examined using the B3LYP functional. Binding energies were calculated to compare the adsorption stability at the basal site versus the edge sulfur site, enabling correlation with the experimental adsorption trends. To represent the GAC sorbent, a graphene-like cluster was employed as a simplified model of the local carbon environment. The surface oxygen functionalities naturally present on activated carbon were represented by a carboxyl group (−COOH) at the cluster edge. This model captures the local chemical features relevant to adsorption without attempting to reproduce the full porous structure of activated carbon. For computational representation, EDT-derived fragments were attached to edge carbon sites to construct simplified sulfur-functionalized carbon models. These models represent plausible local C–S-containing configurations associated with the proposed thiol–ene-type treatment and are not intended to reproduce the full distribution of sulfur species present in the experimental material. Two representative models were constructed (Figure 1): the S-GAC1 model, which contains one EDT chain grafted to the carbon edge, representing isolated sulfur sites; and the S-GAC2 model, which contains two EDT chains, representing a higher local sulfur density with an S–S bridge.

**Figure 1:**
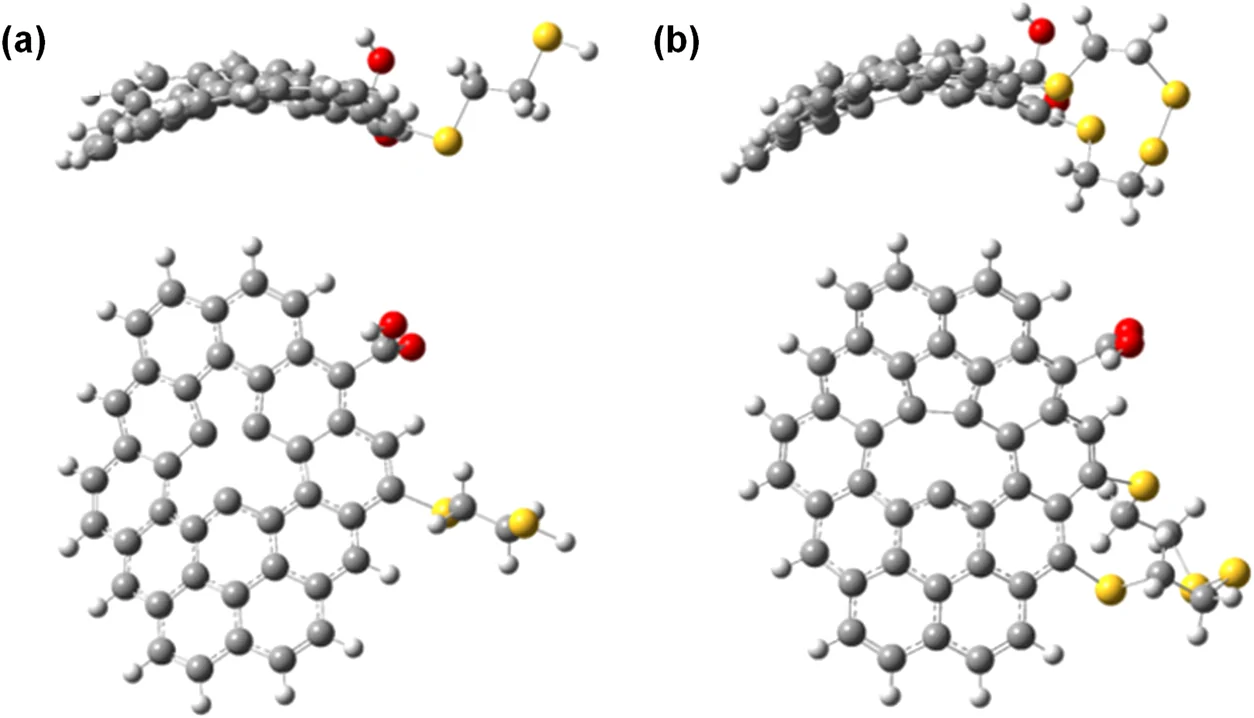
Models of S-GAC used in the DFT calculations: (a) S-GAC1, functionalized with one EDT chain, and (b) S-GAC2, functionalized with two EDT chains. For each model, the top panel shows the top view, whereas the bottom panel shows the corresponding side view. The gray, red, yellow, and white balls represent carbon, oxygen, sulfur, and hydrogen atoms, respectively.

All of the DFT calculations were performed using the Gaussian 16 software package. Geometry optimizations were carried out using the B3LYP exchange-correlation functional. The 6–31+G­(d,p) basis set was applied for C, H, O, S, and Cl, whereas the LANL2DZ effective core potential and basis set were used for Hg. The binding energy (Ebind) of each Hg­(II) species on the model surfaces was calculated according to eq 1

1 $$E_{bind}=E_{\left(S‐\mathrm{GA}C+Hg\;species\right)}-E_{\left(S‐GAC\right)}-E_{\left(Hg\;species\right)}$$

where negative values indicate energetically favorable adsorption. The calculated binding energies and optimized geometries were used to provide molecular-level insight into adsorption behavior, identify preferred binding motifs, and compare the relative affinity of different Hg­(II) species toward isolated versus densely grafted thiol sites on activated carbon.

### 2.6 Stability and Reusability of S-GAC

The chemical stability of S-GAC was evaluated by incubating the samples in aqueous solutions at pH 2.0 ± 0.2, 6.0 ± 0.2, and 10.0 ± 0.2 and adjusting them with 0.1 M HCl or NaOH for 10 days at 25 °C. The recovered materials were analyzed by CHNS elemental analysis to assess sulfur loss. All tests were performed in triplicate. For reusability, 30%S-GAC was subjected to five adsorption–desorption cycles. During each cycle, the adsorbent (1.0 g L^–1^) was in contact with 80 mg L^–1^ Hg­(II) for 24 h. The Hg-loaded sorbent was regenerated with 10.0 mL of 0.1 M HCl, after which it was washed with deionized water until the effluent reached approximately pH 6 and vacuum-dried at room temperature before the next cycle. Reusability tests were performed in triplicate.

## 3 Results and Discussion

### 3.1 Material Formation and Physicochemical Properties

The introduction of EDT-derived sulfur functionalities onto GAC following thermally initiated thiol–ene-type treatment was supported by complementary spectroscopic, elemental, thermal, and textural analyses (Figures 2 and 3). Pristine GAC exhibited characteristic D and G bands at approximately 1350 and 1580 cm^–1^, corresponding to disordered and graphitic carbon domains, respectively.^38,39^ Following functionalization, additional Raman bands appeared at approximately 498 and 698 cm^–1^, which were tentatively assigned to S–S and C–S stretching vibrations, respectively.^40^ Together with the elemental sulfur and XPS results, these features are consistent with the introduction of reduced sulfur functionalities onto the carbon material (Figure 2b).

**Figure 2:**
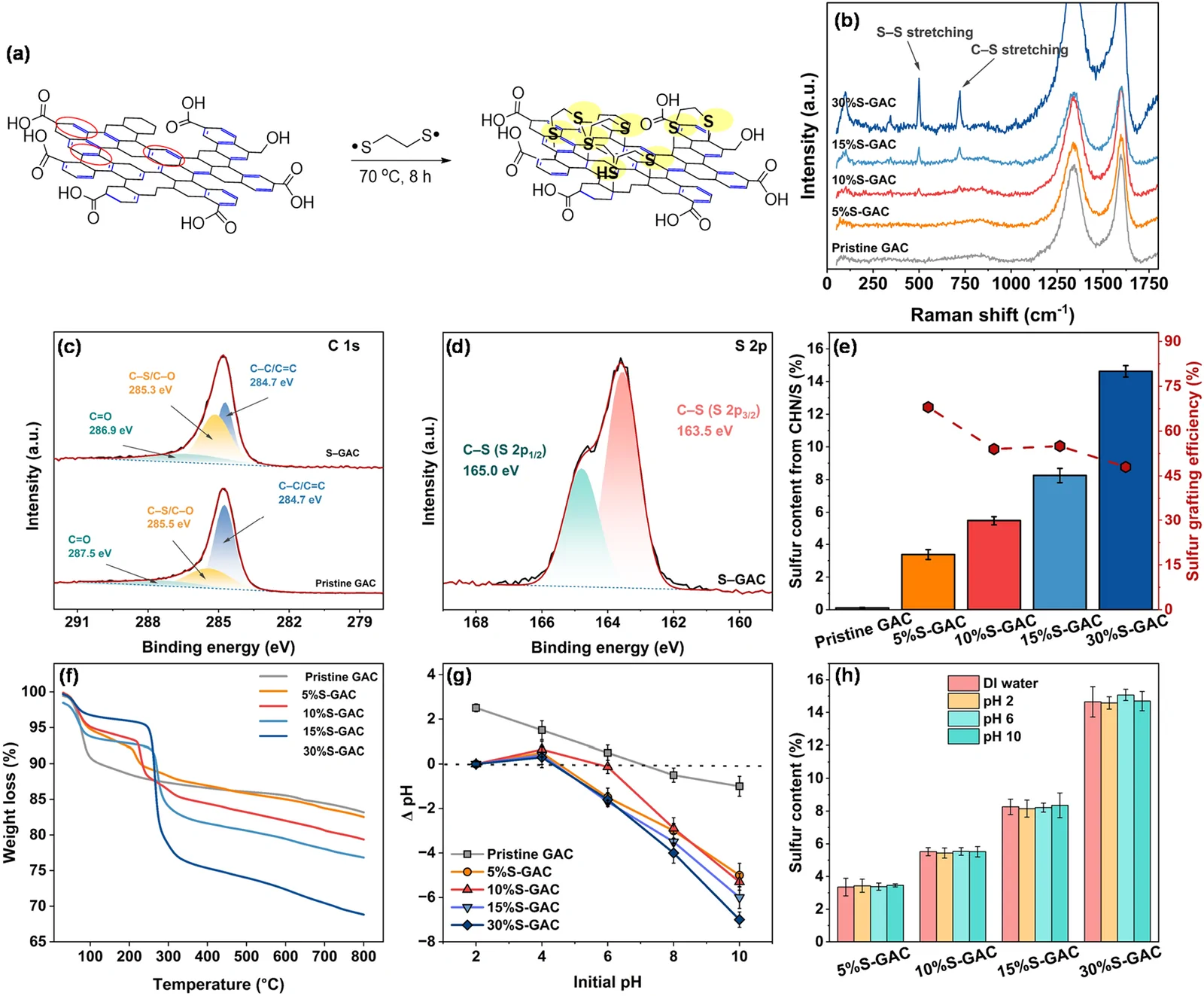
Physicochemical characterization of pristine GAC and S-GAC: (a) proposed oxidant-free, thermally initiated thiol–ene-type functionalization of reactive carbon-surface sites with EDT-derived sulfur functionalities; (b) Raman spectra showing carbon D and G bands and sulfur-associated vibrations; (c) high-resolution C 1s spectra of pristine GAC and S-GAC; (d) S 2p spectrum of S-GAC; (e) measured sulfur content and percentage retention of added sulfur as a function of nominal sulfur loading; (f) TGA profiles; (g) determination of pH_pzc_; and (h) sulfur contents before and after 10 days of exposure at pH 2, 6, and 10.

**Figure 3:**
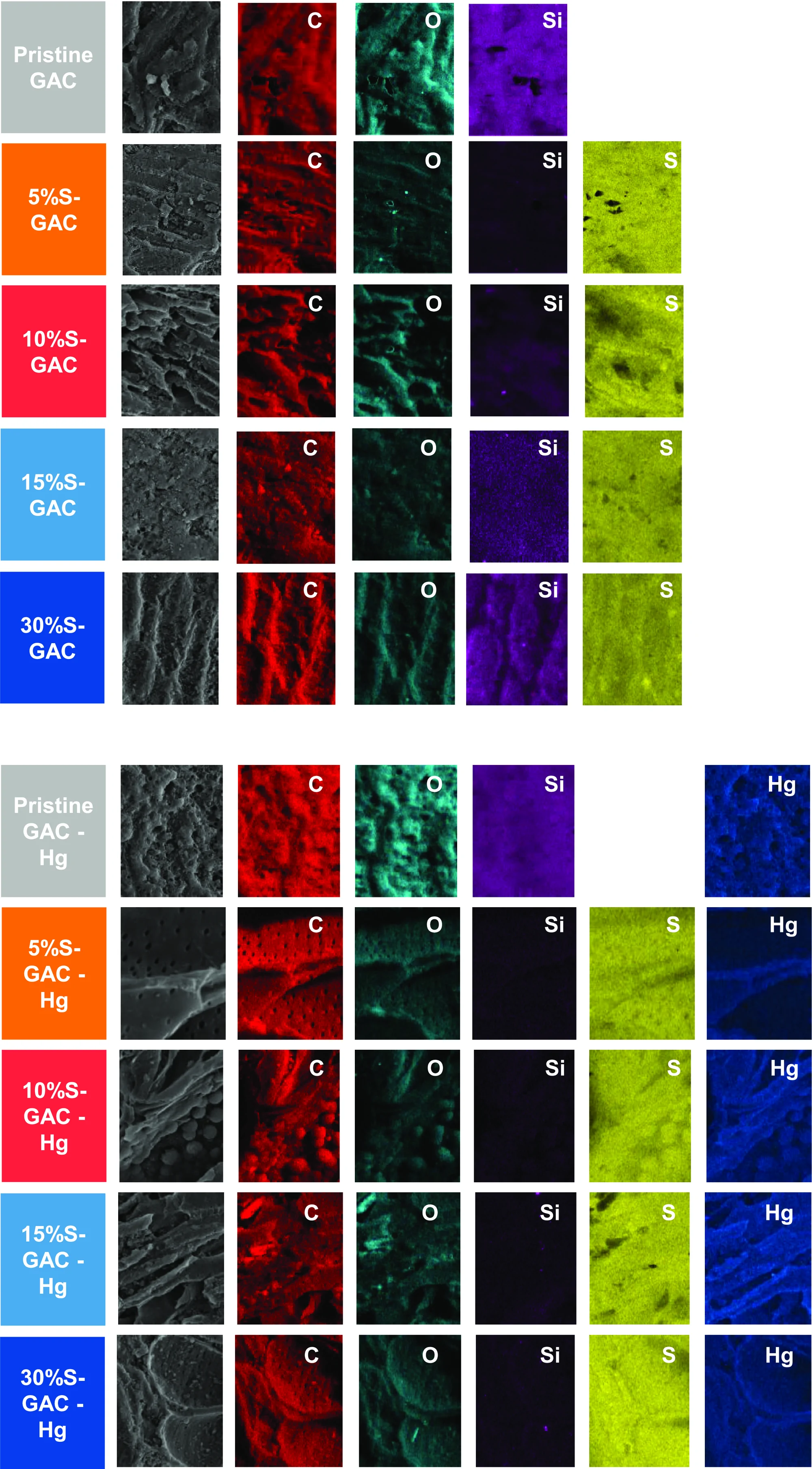
SEM images and corresponding EDS elemental maps of pristine GAC and S-GAC before and after Hg­(II) adsorption.

The XPS results further supported changes in surface composition following sulfur functionalization. In the C 1s spectrum, the relative contribution of the C–C/C=C component decreased from 51.07% for pristine GAC to 27.72% for S-GAC, whereas the overlapping C–S/C–O component increased from 18.00% to 44.47% (Figure 2c and Table 1).^41^ Although the C–S and C–O contributions cannot be fully resolved from the C 1s region alone, this redistribution, together with the S 2p spectrum, supports modification of the carbon surface. The S 2p doublet at 163.5 and 165.0 eV is consistent with reduced sulfur environments, including C–S-, thiol-, and S–S-related species.^42,43^ Because several reduced sulfur configurations may contribute within this binding-energy range, the spectra support sulfur functionalization but do not uniquely establish a single surface structure or reaction pathway. No appreciable signal was observed near 168 eV, indicating that oxidized sulfur species were not a major component of the grafted surface.^44^ The retained reduced sulfur environment is favorable for coordination with the soft Lewis acid Hg­(II).

**Table 1:** Relative Contributions of Carbon Functional Groups Obtained from Deconvolution of the High-Resolution C 1s XPS Spectra of Pristine GAC and S-GAC

	area		% area	
chemical bonding	pristine GAC	S-GAC	pristine GAC	S-GAC
C–C/C=C	7136.8	2868.3	51.07	27.72
C–S/C–O	2515.9	4600.6	18.00	44.47
C=O	4323.5	2877.3	30.93	27.81

Elemental analysis revealed that the sulfur content increased systematically with the nominal sulfur dose (Figure 2e). In contrast, the percentage of added sulfur retained by the carbon decreased as the precursor dose increased, indicating a decrease in the sulfur-retention efficiency at higher loadings. This trend is consistent with a limited number of accessible reactive sites, increasing steric constraints between EDT-derived functionalities, or reduced accessibility of internal carbon surfaces.^45^ Thermal analysis revealed an initial mass loss below approximately 110 °C, attributed primarily to adsorbed moisture, followed by an additional mass loss regions at approximately 200–250 °C associated with the decomposition of the grafted organic sulfur functionalities.^46^ These results indicate that sulfur incorporation altered the thermal profile of GAC and that the incorporated sulfur-containing moieties remained stable well above the adsorption operating temperature. The persistence of sulfur after repeated ethanol washing indicates that the incorporated sulfur species were not readily removed under the washing conditions. However, elemental analysis alone cannot distinguish covalently attached functionalities from strongly retained or cross-linked EDT-derived species.

Sulfur functionalization also shifted the pH_pzc_ from approximately 6.5 for pristine GAC to 4.2–4.3 for the S-GAC samples (Figure 2g), reflecting the change in surface chemistry after grafting.^47^ At an adsorption pH of 6.0, the S-GAC surface was therefore predominantly negatively charged. Electrostatic interactions may contribute to the uptake of positively charged Hg species; however, the strong chemical affinity of reduced sulfur sites for Hg­(II) is expected to remain the principal binding contribution.

The incorporated sulfur functionalities exhibited a high chemical stability across the investigated pH range. After exposure to aqueous solutions at pH 2, 6, and 10 for 10 days, no appreciable change in the bulk sulfur content was detected by CHNS analysis (Figure 2h). These results indicate a minimal loss of incorporated sulfur under acidic, neutral, and alkaline conditions and support the strong retention of sulfur-containing functionalities.^48^ Because sulfur content was measured in the recovered solids rather than directly in the leachates, the result is most appropriately interpreted as evidence of sulfur retention rather than the complete absence of sulfur release. The stability result supports strong retention of the sulfur-containing functionalities but does not independently prove the detailed covalent structure proposed in Figure 2a.

Sulfur grafting substantially altered the textural properties of the GAC (Table 2). Pristine GAC exhibited a BET specific surface area of 859 m^2^ g^–1^, a total pore volume of 0.35 cm^3^ g^–1^, and a micropore volume of 0.30 cm^3^ g^–1^, which are consistent with the highly microporous structure of coconut-shell activated carbon.^48^ The BET specific surface area decreased progressively to 705, 247, and 24 m^2^ g^–1^ for 5%S-GAC, 15%S-GAC, and 30%S-GAC, respectively. The approximately 97% decrease observed for 30%S-GAC, accompanied by the depletion of the measurable micropore volume, is consistent with the progressive occupation or obstruction of micropores by EDT-derived sulfur functionalities.^49,50^ Although structural alteration of carbon cannot be excluded solely from gas-sorption data, the systematic relationship between sulfur loading and loss of accessible porosity strongly supports pore filling and pore blocking as the dominant effects. Importantly, the subsequent Hg­(II) adsorption results show that the substantial loss of accessible porosity did not prevent effective Hg­(II) capture at low initial concentrations, whereas its limiting effect became more evident at elevated Hg­(II) loadings.

**Table 2:** BET Surface Area, Total Pore Volume, Micropore Volume, and Average Pore Diameter of Pristine GAC and S-GAC

sample	S_BET_ (m^2^ g^–1^)	*V*_total_ (cm^3^ g^–1^)	*V*_micro_ (cm^3^ g^–1^)	average pore size (nm)
pristine GAC	859	0.35	0.30	1.65
5%S-GAC	705	0.31	0.23	1.76
10%S-GAC	437	0.29	0.13	2.27
15%S-GAC	247	0.12	0.05	3.45
30%S-GAC	25	0.04	0.00	4.50

### 3.2 Time-Dependent Hg­(II) Removal at Low Initial Concentration

The time-dependent decrease in the residual Hg­(II) concentration during adsorption by pristine GAC and S-GAC in the aqueous solution and synthetic gas condensate is shown in Figure 4. At the low initial Hg­(II) concentration used in these experiments (*C*_0_ ≈ 1.18 mg L^–1^) in the aqueous system, sulfur functionalization progressively enhanced Hg­(II) removal, particularly during the early and intermediate contact periods.

**Figure 4:**
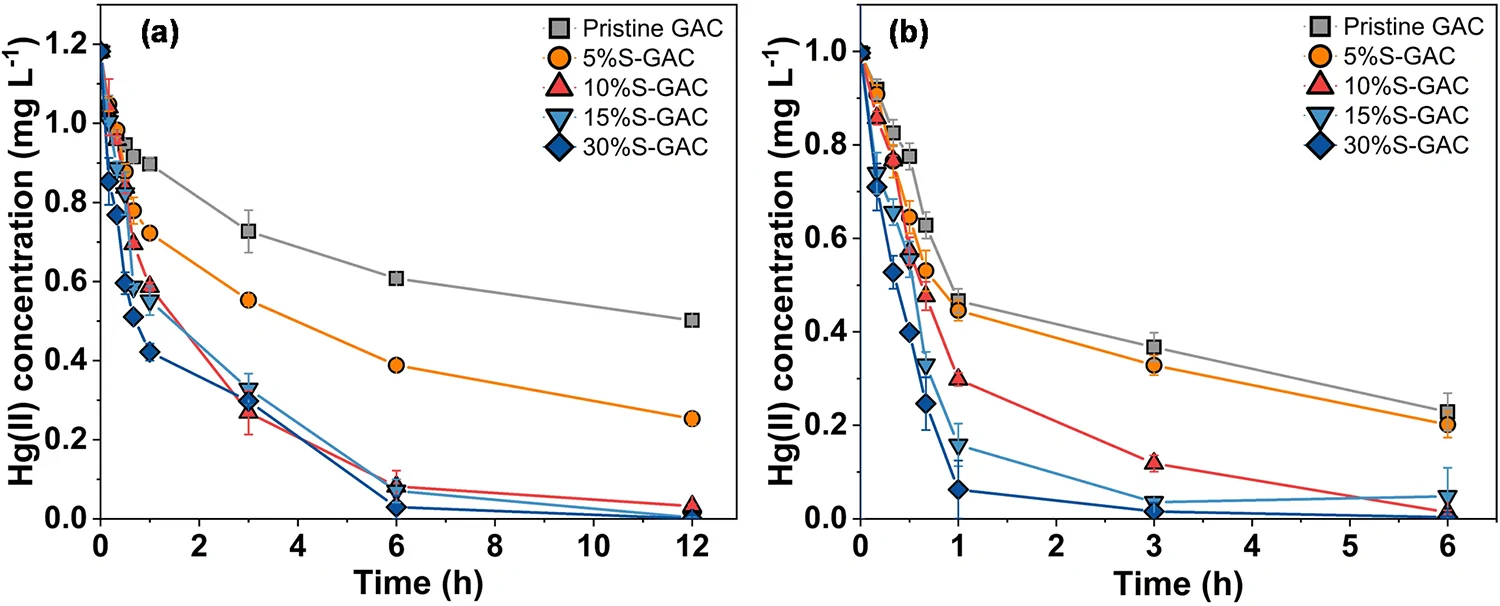
Changes in residual Hg­(II) concentration during adsorption by pristine GAC and S-GAC in (a) aqueous solution over 12 h and (b) synthetic gas condensate over 6 h.

In aqueous solution, the residual Hg­(II) concentration after 1 h decreased from approximately 0.90 mg L^–1^ for pristine GAC to 0.72, 0.59, 0.55, and 0.42 mg L^–1^ for 5%S-GAC, 10%S-GAC, 15%S-GAC, and 30%S-GAC, respectively. This corresponds to an increasing removal efficiency with sulfur loading during the initial stage of adsorption. After 6 h, the residual Hg­(II) concentration remained relatively high for pristine GAC (approximately 0.61 mg L^–1^), whereas substantially lower concentrations were observed for the sulfur-functionalized materials. By 12 h, the residual Hg­(II) concentration had decreased to approximately 0.50 mg L^–1^ for pristine GAC and 0.25 mg L^–1^ for 5%S-GAC, while 10%S-GAC, 15%S-GAC, and 30%S-GAC decreased the concentration of Hg­(II) to approximately 0.032, 0.003, and less than 0.001 mg L^–1^, respectively.

The improved low-concentration performance of S-GAC is consistent with the strong affinity of Hg­(II) for the sulfur-containing binding environments. However, increasing the nominal sulfur loading beyond 10% resulted in progressively smaller improvements in final Hg­(II) removal because the removal rate of 10%S-GAC, 15%S-GAC, and 30%S-GAC approached near-complete after prolonged contact. This suggests that under low-Hg­(II) conditions, sulfur incorporation primarily enhances the rate and affinity of Hg­(II) capture, whereas the benefit of further increasing sulfur loading becomes less pronounced as the residual Hg­(II) concentration approaches very low levels.

### 3.3 Extended-Time Hg­(II) Adsorption at Elevated Initial Concentrations

To determine how sulfur loading influences Hg­(II) adsorption under high loading conditions and long contact times, extended-time experiments were conducted in the aqueous solution at initial Hg­(II) concentrations of approximately 40 and 120 mg L^–1^ (Figure 5). A markedly different sulfur-loading dependence was observed from that obtained at the low initial concentration described in Figure 4. At both elevated concentrations, pristine GAC exhibited the most rapid initial Hg­(II) uptake and approached a relatively stable residual concentration earlier than the sulfur-functionalized materials. In contrast, S-GAC showed slower but sustained adsorption over prolonged contact times, indicating a pronounced effect of sulfur functionalization on adsorption kinetics as well as on the final observed uptake.

**Figure 5:**
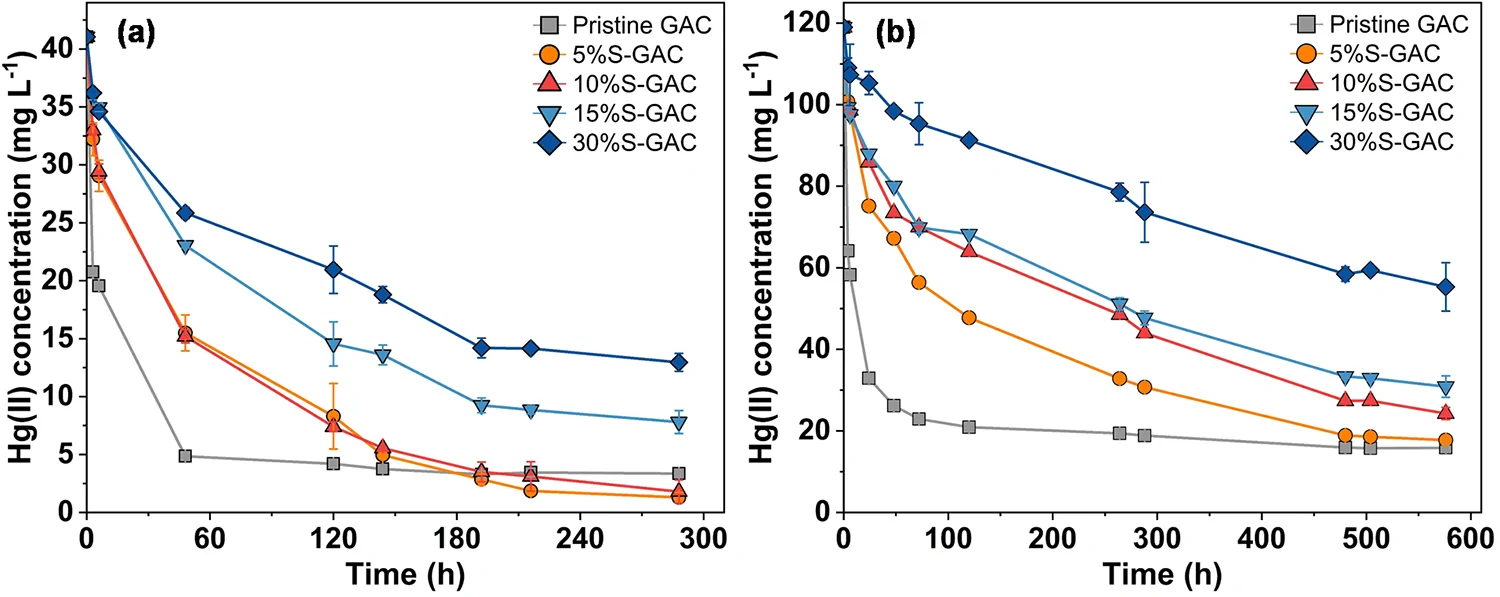
Changes in residual Hg­(II) concentration during extended adsorption by pristine GAC and S-GAC in aqueous solution at initial Hg­(II) concentrations of approximately (a) 40 mg L^–1^ and (b) 120 mg L^–1^. The experiments were monitored for up to 288 and 576 h, respectively.

At an initial Hg­(II) concentration of 41.05 mg L^–1^, pristine GAC rapidly decreased the residual Hg­(II) concentration and approached a relatively stable level during prolonged contact. In contrast, the S-GAC samples continued to remove Hg­(II) gradually throughout the monitored period. By 288 h, the experimental Hg­(II) uptakes were 37.76, 39.98, 38.72, 32.91, and 28.18 mg g^–1^ for pristine GAC, 5%S-GAC, 10%S-GAC, 15%S-GAC, and 30%S-GAC, respectively (Table 3). Notably, the sustained uptake of 5%S-GAC and 10%S-GAC allowed their final observed uptake to slightly exceed that of pristine GAC despite their slower initial adsorption. This behavior demonstrates that the relative performance of pristine and sulfur-functionalized GAC depends not only on Hg­(II) loading but also on contact time.

**Table 3:** Final Monitored Residual Hg­(II) Concentrations and Corresponding Experimental Hg­(II) Uptake by Pristine GAC and S-GAC during Extended-Time Aqueous Adsorption Experiments at Initial Hg­(II) Concentrations of Approximately 40 and 120 mg L^–1^ ^a^

sample	initial Hg(II) concentration (mg L^–1^)	final monitored time (h)	residual Hg(II) concentration, *C*_t_ (mg L^–1^)	experimental Hg(II) uptake at final time, *q*_t_ (mg g^–1^)
pristine GAC	41.05	288	3.35	37.7642
5%S-GAC	41.05	288	1.30	39.9785
10%S-GAC	41.05	288	1.80	38.7157
15%S-GAC	41.05	288	7.80	32.9078
30%S-GAC	41.05	288	12.95	28.1761
pristine GAC	119.0	576	15.85	102.4393
5%S-GAC	119.0	576	17.75	100.8529
10%S-GAC	119.0	576	24.25	94.7204
15%S-GAC	119.0	576	30.85	87.4125
30%S-GAC	119.0	576	55.30	63.5134

a *q*_t_ was calculated from the measured initial and final Hg­(II) concentrations using *q*_t_ = (*C*_0_ – *C*_t_)­V/m, where *C*_0_ and *C*_t_ are the initial and final Hg­(II) concentrations, respectively, *V* is the solution volume, and *m* is the adsorbent mass. The reported values represent uptake at the final monitored time and should not be interpreted as equilibrium adsorption capacities.

A similar distinction between rapid uptake by pristine GAC and prolonged adsorption by S-GAC was observed at an initial Hg­(II) concentration of 119.0 mg L^–1^. By 576 h, the experimental Hg­(II) uptakes were 102.44, 100.85, 94.72, 87.41, and 63.51 mg g^–1^ for pristine GAC, 5%S-GAC, 10%S-GAC, 15%S-GAC, and 30%S-GAC, respectively (Table 3). Thus, pristine GAC retained the highest observed uptake at this higher Hg­(II) loading, although 5%S-GAC progressively approached a comparable value. The decrease in residual Hg­(II) concentration for the S-GAC samples at the end of the monitoring period indicates that their adsorption had not yet reached a clear plateau. Pristine GAC approached an apparent plateau by approximately 192 and 480 h at initial Hg­(II) concentrations of approximately 40 and 120 mg L^–1^, respectively, whereas none of the S-GAC samples reached a clear plateau within the corresponding monitoring periods of 288 and 576 h.

The contrasting behavior between the low- and high-concentration experiments suggests that sulfur functionalization changes the relative importance of the processes controlling Hg­(II) adsorption. At low-Hg­(II) loading, the strong affinity of sulfur-containing sites for Hg­(II) provides a clear advantage, resulting in rapid removal and very low residual Hg­(II) concentrations. At higher Hg­(II) loadings, however, the contribution of the porous carbon structure becomes increasingly important. Sulfur functionalization substantially decreases surface area and micropore accessibility, and at high sulfur loadings, these structural penalties may outweigh the benefit of introducing additional high-affinity sulfur donor sites. Similar trade-offs between sulfur functionality and the loss of accessible pore structure have been reported for sulfur-modified activated carbons.^51,52^ These results indicate that the affinity-accessibility trade-off is also time dependent. The highly porous pristine GAC provides rapid access to adsorption sites and therefore dominates the initial uptake at elevated Hg­(II) loading, whereas sulfur-functionalized GAC exhibits slower, sustained adsorption that can partially compensate for its reduced pore accessibility over a prolonged contact.

The prolonged decrease in the residual Hg­(II) concentration observed for the S-GAC samples further suggests that the incorporated sulfur-containing domains are not accessed instantaneously. Instead, Hg­(II) uptake may increasingly depend on slower intraparticle transport and diffusion into restricted pore regions as the adsorption proceeds. This interpretation is consistent with the substantial decrease in the BET surface area and micropore volume observed with increasing sulfur loading. However, the present data do not distinguish quantitatively between diffusion limitations and differences in the accessibility or reactivity of individual sulfur-containing sites.

Taken together with the low-concentration results shown in Figure 4, these findings demonstrate that sulfur functionalization does not simply increase Hg­(II) adsorption across all loading conditions. Rather, its effect depends strongly on the Hg­(II) concentration and the balance between local Hg–S affinity and the accessibility of the porous carbon structure. Favorable Hg–S coordination at the molecular scale therefore does not necessarily translate into superior macroscopic adsorption performance at elevated Hg­(II) loading. Overall Hg­(II) capture is governed by the combined effects of sulfur-site affinity, pore accessibility, mass transfer, Hg­(II) loading, and contact time.

### 3.4 Time-Dependent Hg­(II) Adsorption in Synthetic Gas Condensate

The time-dependent Hg­(II) adsorption behavior in synthetic gas condensate is shown in Figure 4b. At the low initial Hg­(II) concentration used in this experiment (*C*_0_ = 0.997 mg L^–1^), sulfur functionalization generally accelerated Hg­(II) removal from the hydrocarbon matrix, and the effect became more pronounced with increasing sulfur loading during the early stages of adsorption.

After 1 h, the residual Hg­(II) concentration decreased to approximately 0.47 and 0.45 mg L^–1^ for pristine GAC and 5%S-GAC, respectively, whereas substantially lower concentrations were observed for 10%S-GAC, 15%S-GAC, and 30%S-GAC (approximately 0.30, 0.16, and 0.063 mg L^–1^, respectively). These results indicate that the benefit of sulfur functionalization was most evident at higher sulfur loadings during the initial adsorption period.

At longer contact times, the differences among the higher-sulfur materials became less pronounced. After 6 h, the residual Hg­(II) concentrations were approximately 0.23 and 0.20 mg L^–1^ for pristine GAC and 5%S-GAC, respectively, while 10%S-GAC and 30%S-GAC reduced Hg­(II) to very low residual concentrations of approximately 0.014 and 0.003 mg L^–1^, respectively. The mean residual concentration for 15%S-GAC was approximately 0.048 mg L^–1^, although greater variability between replicates was observed at this time point.

These results demonstrate that sulfur-containing functionalities substantially increase the level of Hg­(II) removal from the nonpolar condensate matrix under low-concentration conditions. The stronger performance of the higher-sulfur materials is consistent with the high affinity of soft sulfur donor sites toward Hg species and with the sulfur-containing Hg coordination environments identified by spectroscopic analyses in this study. However, the small differences among 10%S-GAC, 15%S-GAC, and 30%S-GAC at longer contact times indicate that further increasing the sulfur loading decreased the practical benefit once very low residual Hg­(II) concentrations were approached.

The condensate-phase behavior also illustrates the importance of the treatment matrix in controlling adsorption performance. Differences in Hg solvation, wetting of the carbon surface, mass transfer, and accessibility of sulfur-containing domains between aqueous and hydrocarbon media may influence the relative contribution of sulfur affinity and pore accessibility.^53,54^ Accordingly, sulfur loading should not be considered independent of the matrix or concentration regime when evaluating the S-GAC performance.

### 3.5 DFT Insights into Hg­(II) Adsorption

To elucidate the mechanism of Hg­(II) adsorption on S-GAC, DFT calculations were performed using representative S-GAC1 and S-GAC2 models (Figure 1). Under the experimental conditions (pH 6), Hg­(II) is expected to exist predominantly as neutral HgCl_2_, while HgCl^+^ is a minor species, and Hg^2+^ is negligible.^55–57^ Therefore, HgCl_2_ was selected as the primary adsorption model to rationalize the experimentally observed strong Hg­(II) uptake of S-GAC, while calculations for HgCl^+^ are provided in Figure S2 for comparison.

The adsorption of HgCl_2_ on different regions (top vs edge) of S-GAC1 and S-GAC2 is compared in Figure 6. Within the simplified models examined, HgCl_2_ binding was more favorable near sulfur-containing edge configurations than on the basal carbon region. For the S-GAC1 model, the calculated binding energy at the edge site was more negative than that at the basal site, indicating stronger stabilization near the EDT-derived sulfur functionality. This trend is consistent with the hard and soft acids and bases principle, in which Hg­(II) acts as a soft acid and sulfur donor sites act as soft bases. The calculations therefore provide a qualitative molecular rationale for the experimentally observed Hg affinity of sulfur-functionalized GAC. The calculated binding energies are −22.85 kcal mol^–1^ for the basal adsorption (Figure 6a) and −25.12 kcal mol^–1^ for the edge configuration (Figure 6b). The more negative value at the edge indicates more favorable stabilization near sulfur-containing configurations than on the basal surface. With respect to S-GAC2, which has a relatively high local sulfur density, HgCl_2_ stabilized even more strongly at edge sites. The binding energies are −25.26 kcal mol^–1^ for basal adsorption (Figure 6c) and −35.98 kcal mol^–1^ for edge adsorption (Figure 6d). Importantly, the stronger adsorption in S-GAC2 than in S-GAC1 is consistent with a possible multidentate/chelation-like interaction. The DFT-predicted preference for sulfur-rich edge configurations is qualitatively consistent with the Hg 4f XPS results and the sulfur-containing coordination environments supported by the EXAFS analysis.^42,44^

**Figure 6:**
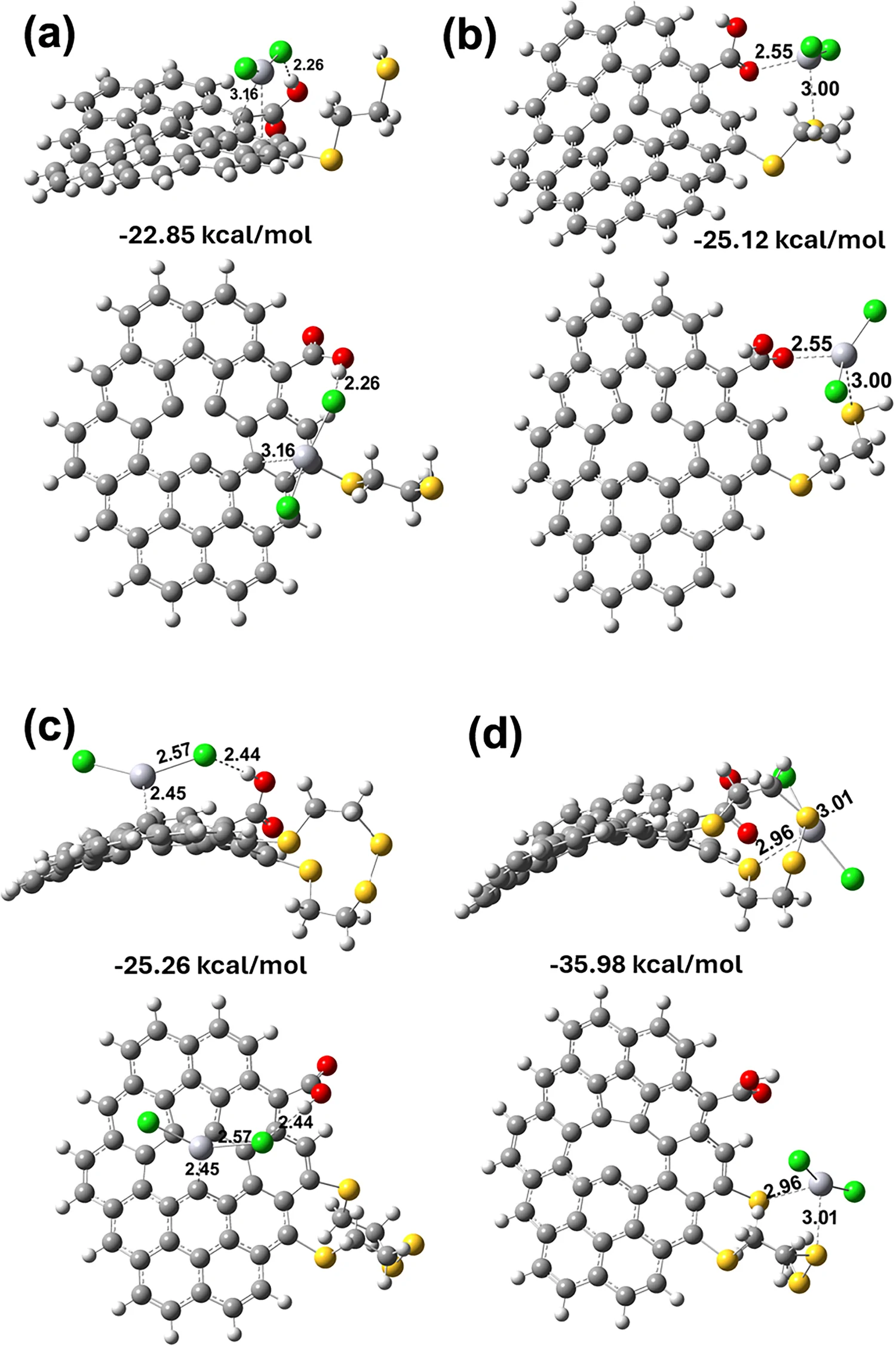
Adsorption configuration and binding energy (kcal mol^–1^) of HgCl_2_ on (a) the top of S-GAC1, (b) the edge of the S-GAC1 model, (c) the basal region of S-GAC2, and (d) the edge of the S-GAC2 model. For each model, the top panel shows the top view, whereas the bottom panel shows the corresponding side view. The gray, red, yellow, green, and white balls represent carbon, oxygen, sulfur, chlorine, and hydrogen, respectively.

### 3.6 Hg–S Binding Mechanism

The molecular origin of the strong Hg­(II) affinity of S-GAC was investigated using complementary XPS, SEM–EDS, and Hg L_3_-edge XAS analyses. The high-resolution Hg 4f spectrum of the Hg-loaded S-GAC exhibited characteristic Hg 4f_7/2_ and Hg 4f_5/2_ doublets at approximately 100.4 and 104.4 eV, respectively (Figure 7a).^42,44^ These binding energies are consistent with those of Hg coordinated to reduced sulfur environments, including sulfide- or thiolate-like Hg–S species, and support chemical binding rather than solely nonspecific physical adsorption. SEM–EDS mapping further revealed that Hg was distributed over sulfur-containing regions of 30%S-GAC after adsorption (Figure 3), which is consistent with the incorporated sulfur functionalities contributing to Hg retention.^41^ Because EDS provides spatial co-occurrence rather than direct evidence of bonding, the local coordination environment was further examined by XAS.

**Figure 7:**
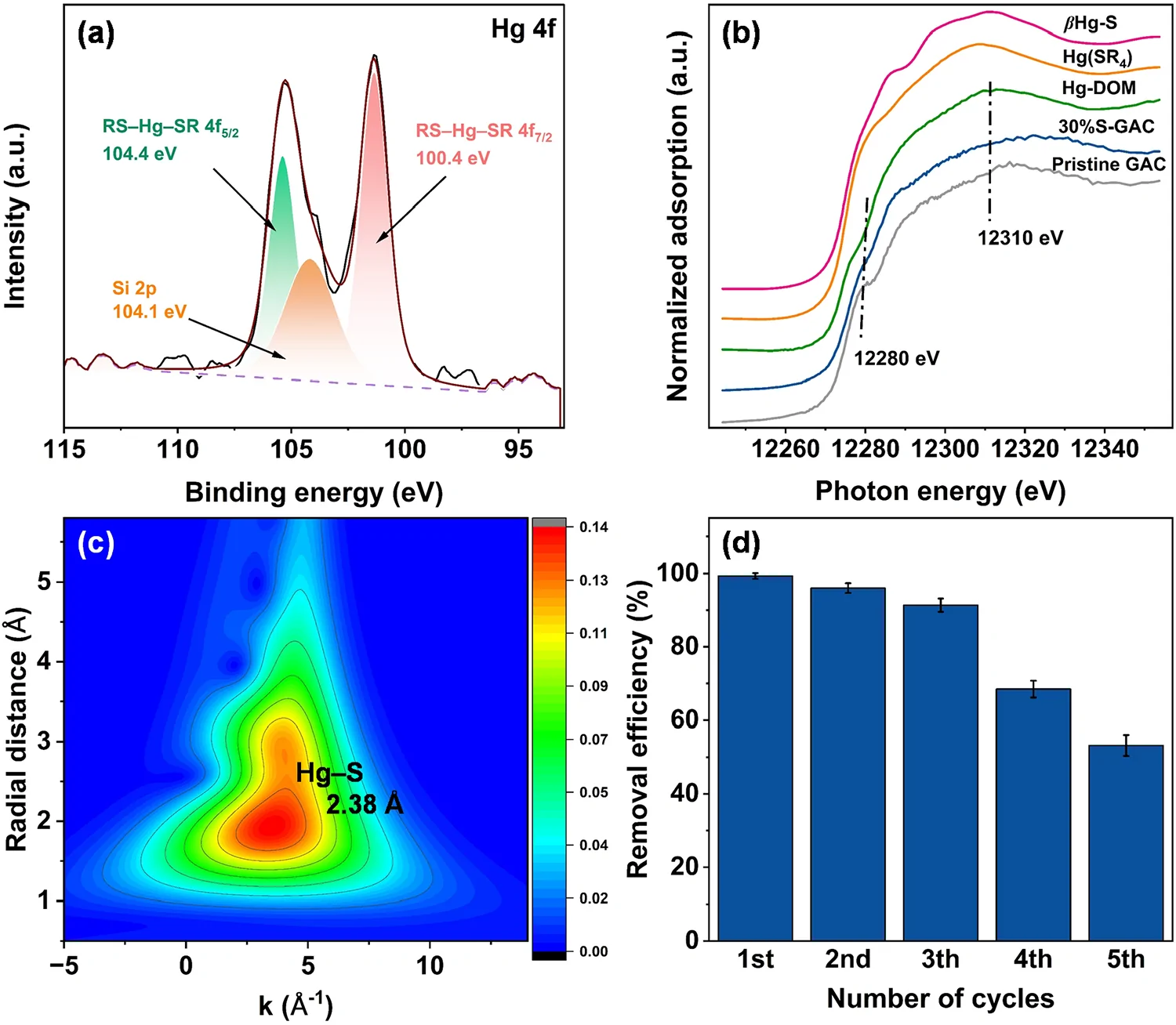
Spectroscopic characterization of Hg binding and reusability of S-GAC: (a) high-resolution Hg 4f XPS spectrum of Hg-loaded S-GAC, consistent with Hg–S coordination; (b) normalized Hg L_3_-edge XANES spectra of Hg-loaded GAC and S-GAC compared with selected Hg reference spectra; (c) wavelet-transform analysis of the Hg L_3_-edge EXAFS signal showing a scattering feature consistent with Hg–S coordination; and (d) Hg­(II) removal by 30%S-GAC over five adsorption–desorption cycles.

The normalized Hg L_3_-edge XANES spectra of the Hg-loaded pristine GAC and 30%S-GAC were evaluated by linear-combination fitting using β-HgS, Hg­(SR)_4_, and Hg–DOM as reference spectra (Figure 7b and Table S1). For GAC-Hg, the fitted contributions were 6.1% β-HgS, 40.0% Hg­(SR)_4_, and 53.9% Hg–DOM, whereas contributions of 30%S-GAC-Hg were 5.2%, 25.0%, and 69.8%, respectively. The relatively small β-HgS-like contribution in both samples indicates that their XANES spectra more closely resemble the selected organic Hg reference environments than crystalline β-HgS.^58^ However, because the selected reference spectra do not uniquely represent individual coordination structures, these fractions are interpreted as measures of spectral similarity rather than direct quantitative speciation. Wavelet-transform analysis of the Hg L_3_-edge EXAFS signal further revealed a scattering feature consistent with Hg–S coordination at an apparent radial distance of approximately 2.38 Å (Figure 7c).^59,60^ Taken together, the Hg 4f XPS and EXAFS results support the retention of Hg in sulfur-containing coordination environments on S-GAC, whereas XANES-LCF analysis provides complementary information on the local electronic structure.

These experimental observations are also consistent with the DFT results presented in Figure 6. The calculations predict more favorable HgCl_2_ adsorption at sulfur-rich edge sites than that on the basal carbon surface, with the strongest stabilization obtained for the high-local-density sulfur configuration. Thus, the local Hg affinity of S-GAC depends not only on the sulfur content but also on the proximity, accessibility, and local arrangement of the sulfur donor sites.

### 3.7 Sulfur Retention and Regeneration Limitations

The operational durability of 30%S-GAC was assessed over five adsorption–desorption cycles using dilute HCl as the regenerant (Figure 7d). Hg­(II) removal remained above approximately 90% during the first three cycles but decreased to approximately 68% and 53% in the fourth and fifth cycles, respectively. This decrease indicates that acid treatment restored only part of the adsorption capacity and that regeneration became progressively less effective with repeated use. The decline in performance is consistent with incomplete desorption of strongly coordinated Hg–S species, which would progressively occupy otherwise available sulfur sites.^44^ This interpretation is consistent with the spectroscopic evidence for Hg–S coordination and the DFT-predicted preference for multidentate sulfur-rich binding environments. However, because sulfur content was not measured after each adsorption–desorption cycle, the capacity loss cannot be attributed exclusively to persistent Hg occupation; incomplete desorption, pore obstruction, or changes in site accessibility may also contribute.

Independent pH stability tests revealed that the sulfur content of S-GAC remained essentially unchanged after 10 days at pH 2–10 (Figure 2h), indicating strong sulfur retention across the investigated pH range. The combination of sulfur retention and partial reusability supports the durability of the sulfur-functionalized material, while the observed capacity loss emphasizes the need to optimize regeneration conditions that balance Hg­(II) recovery with the preservation of sulfur-containing functionalities.

## 4 Conclusions

An oxidant-free thiol–ene-type treatment introduced sulfur functionalities onto GAC but progressively reduced pore accessibility with increasing sulfur loading. Sulfur functionalization enhanced Hg­(II) removal at low initial concentrations in both aqueous solution and synthetic gas condensate. At elevated aqueous Hg­(II) concentrations, pristine GAC exhibited faster initial uptake and approached a plateau earlier, whereas S-GAC showed slower but sustained adsorption over prolonged contact times. At approximately 40 mg L^–1^, moderate sulfur loadings (5–10%S-GAC) ultimately achieved slightly greater observed uptake than pristine GAC, while pristine GAC remained superior over the monitored period at approximately 120 mg L^–1^. These results show that Hg­(II) capture is governed by the interplay among Hg–S affinity, pore accessibility, Hg­(II) loading, and contact time rather than by sulfur content alone. XPS, Hg L_3_-edge XAS, and DFT calculations were consistent with sulfur-containing Hg coordination environments, while the incorporated sulfur remained strongly retained from pH 2–10. From an application perspective, these findings indicate that adsorbent selection and sulfur loading should be matched not only to the expected Hg­(II) concentration but also to the available contact time. S-GAC may be particularly advantageous for low-concentration polishing or processes allowing prolonged contact, whereas pristine or lightly functionalized GAC may be preferable when rapid bulk removal is required.

## Supplementary Material



## Notes

EDS spectra and semiquantitative elemental compositions of pristine and sulfur-functionalized GAC before and after Hg­(II) adsorption; additional DFT adsorption configurations and binding energies for HgCl^+^ on S-GAC models; and Hg L_3_-edge XANES linear-combination fitting results (PDF)
