# Supplementary material for: Oxidant-Free Thiol–Ene Grafting of Granular Activated Carbon for Mercury(II) Adsorption from Aqueous Solution and Synthetic Gas Condensate
Source: ACS Omega. 2026 Oct 26;11(44):69241–54. doi: 10.1021/acsomega.6c09402 (PMC13756672; doi:10.1021/acsomega.6c09402)
Supplement: Supplementary file 1 [file ao6c09402_si_001.pdf]

Supporting Information

Oxidant-Free Thiol–Ene Grafting of Granular Activated Carbon for Mercury(II) Adsorption from Aqueous Solution and Synthetic Gas Condensate

Aphinan Saengsrichan[1,2], Jakkapop Phanthasri[3], Supawadee Namuangruk[3], Poobodin Mano[3], Suttipong Wannapaiboon[4], Doan Pham Minh[5,6], Sanchai Kuboon[3], Saran Youngjan[3], Teera Butburee[3], Issaraporn Rakngam[3], Panot Krukkratoke[3], Gopinathan Sankar[7], Nurak Grisdanurak[8], Suthirat Kittpongvises[6], Rattabal Khunphonoi[9], Pongtanawat Khemthong[3,], Pummarin Khamdahsag[6,10,*]*

[1]International Program in Hazardous Substance and Environmental Management, College of Interdisciplinary and Integrative Studies, Chulalongkorn University, Bangkok 10330, Thailand

[2]Center of Excellence on Hazardous Substance Management (HSM), Chulalongkorn University, Bangkok 10330, Thailand

[3]National Nanotechnology Center (NANOTEC), National Science and Technology Development Agency (NSTDA), Pathum Thani 12120, Thailand

[4]Synchrotron Research and Applications Division, Synchrotron Light Research Institute (Public Organization), Nakhon Ratchasima 30000, Thailand

[5]Centre RAPSODEE, IMT Mines Albi, UMR CNRS 5302, Université de Toulouse, 81013 Albi Cedex 9, France

[6]Sustainable Environment Research Institute, Chulalongkorn University, Bangkok 10330, Thailand

[7]Department of Chemistry, University College London, London, WC1H 0AJ, United Kingdom

[8]Center of Excellence in Environmental Catalysis and Adsorption, Faculty of Engineering, Thammasat University, Pathum Thani 12121, Thailand

[9]Department of Environmental Engineering, Faculty of Engineering, Khon Kaen University, Khon Kaen 40002, Thailand

[10]Hub of Waste Management for Sustainable Development, Center of Excellence on Hazardous Substance Management, Chulalongkorn University, Bangkok 10330, Thailand

*Corresponding authors: pongtanawat@nanotec.or.th (P. Khemthong) and pummarin.k@chula.ac.th (P. Khamdahsag)

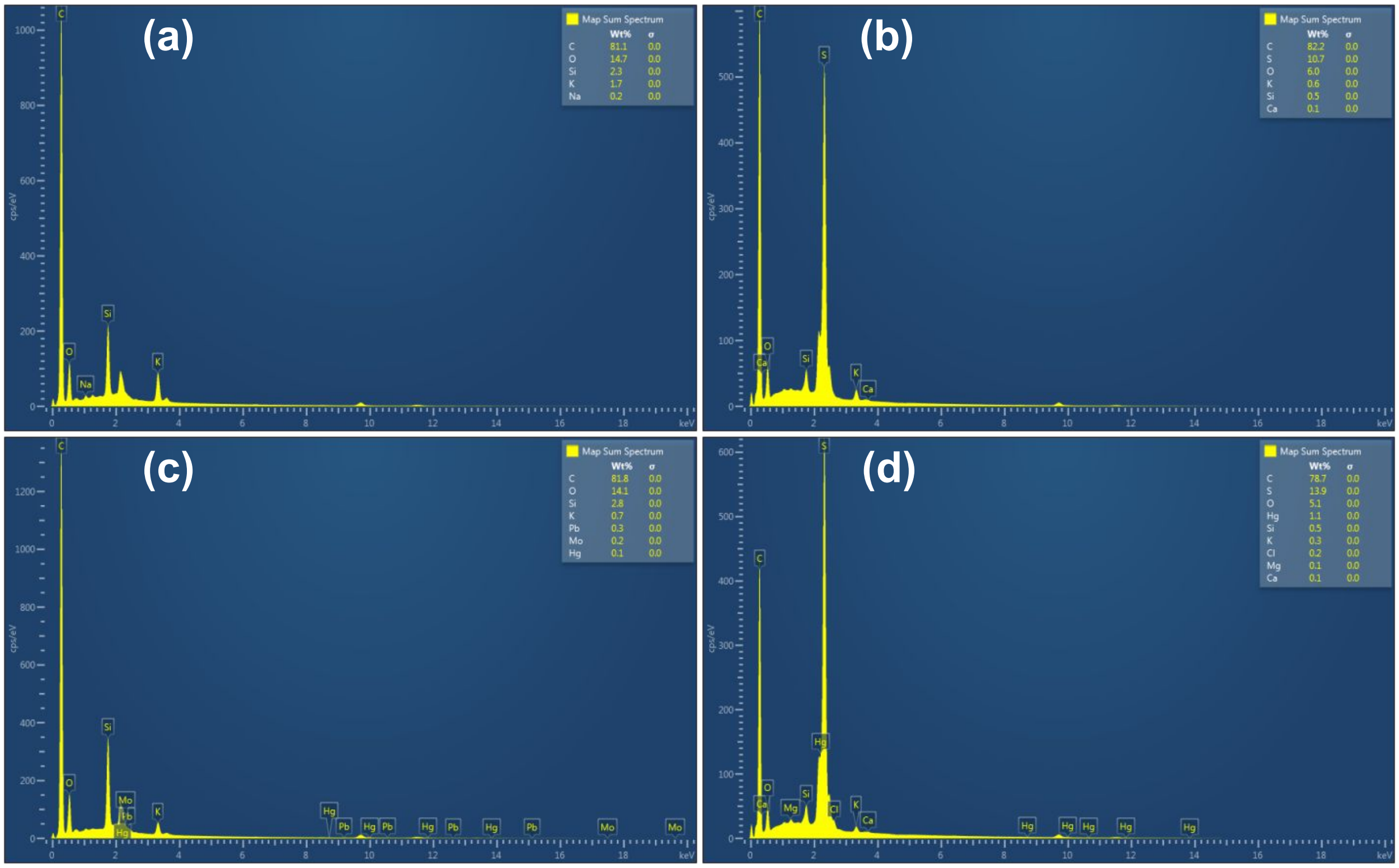

Figure S1. EDS spectra and corresponding semiquantitative elemental compositions (wt%) of pristine GAC and 30%S-GAC before and after Hg(II) adsorption: (a) pristine GAC, (b) 30%S-GAC, (c) Hg-loaded pristine GAC, and (d) Hg-loaded 30%S-GAC.

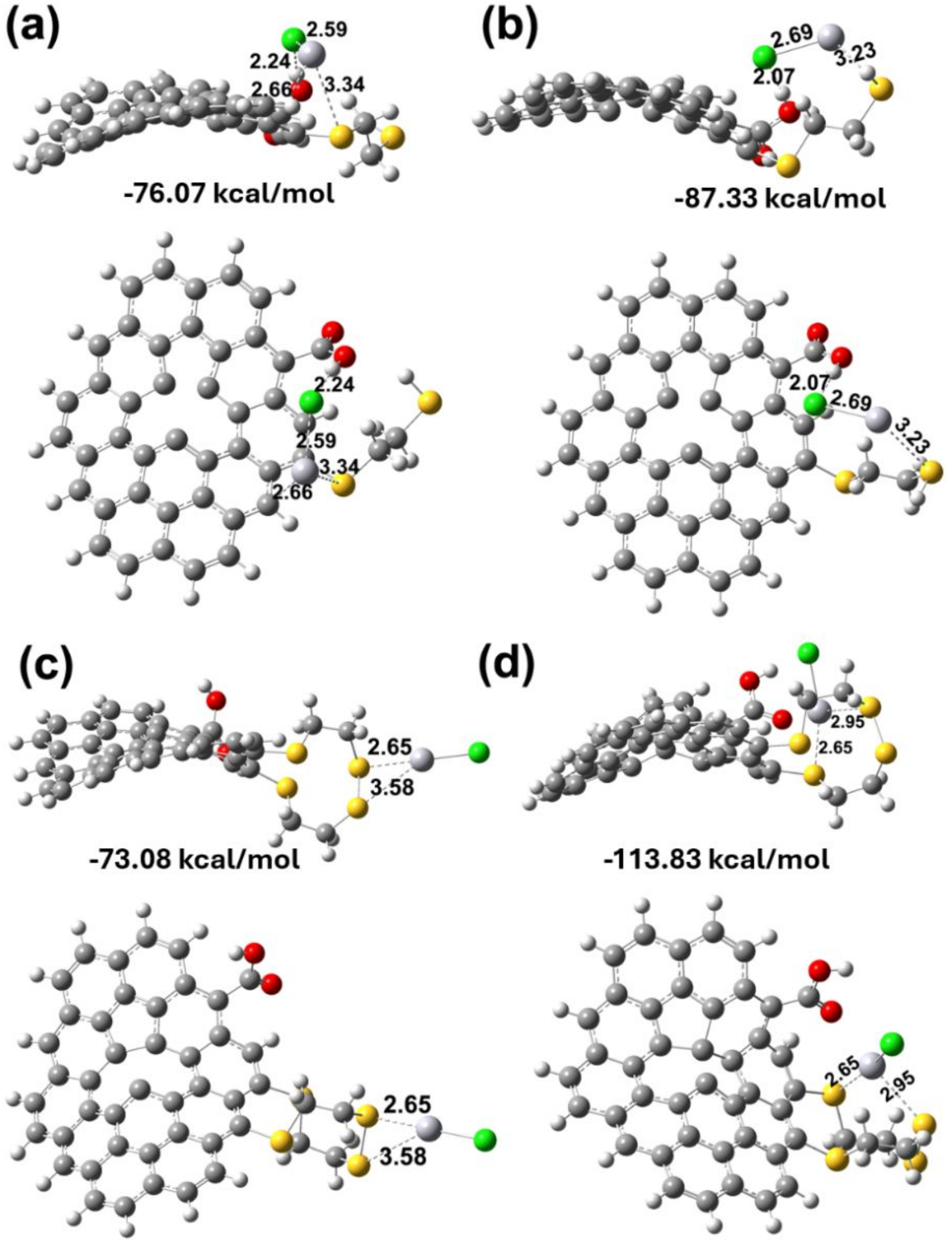

Figure S2. Adsorption configuration and binding energy (kcal mol^{-1}) of $HgCl^+$ on (a) the top of S-GAC1, (b) the edge of S-GAC1 model, (c) the S-S bridge of S-GAC2, and (d) the edge of S-GAC2 models. The results show that $HgCl^+$ adsorption was strongest at the high-density edge sulfur configuration of S-GAC2.

Table S1. Linear combination fitting results of the Hg L_3-edge XANES spectra for Hg-loaded pristine GAC and 30%S-GAC using β-HgS, $Hg(SR)_4$, and Hg–DOM as reference compounds.

Sample	Weight fraction (%)			R-factor	Reduced chi-square
	β-HgS	Hg-$(SR)_4$	Hg–DOM		
GAC-Hg	0.061 (0.006)	0.400 (0.061)	0.539 (0.053)	0.0075	0.0008
30%S-GAC-Hg	0.052 (0.004)	0.250 (0.031)	0.698 (0.031)	0.0024	0.0003

Note:
1. Hg L_3-edge given the energy range (12255.7 to 12305.7 eV).
2. Linear-combination fitting was performed between −20 and +30 eV.
